# A comparative clinical study of stress distribution around implant supporting all - on - four versus all - on - 6 prosthetic concepts

**DOI:** 10.6026/973206300221034

**Published:** 2026-02-28

**Authors:** Pudi Sriharsha, Vivek Anne, Tejosmita Chowdary Pavuluri, Nitish Tyagi, Ranishree Behera, Sreeja Gummalla, Vikas Singh

**Affiliations:** 1 Department of Prosthodontics and Crown & Bridge, MNR Dental College and Hospital, Sangareddy, Telangana, India; 2 Department of Epidemiology, MPH(Epidemiology), Laramie Pediatrics & Internal Medicine pc, 1252 N 22nd St Unit B, Laramie, WY 82072; 3 Department of Biomedical Tejosmita Chowdary Pavuluri, BDS, M.S. Biomedical Sciences (Dental Scholars Track) Candidate, Sciences, Rutgers Biomedical and Health Sciences, Newark, New Jersey, 07107, USA; 4 Department of Periodontology, Family Dental Care, Delhi Dental Council, Delhi, India; 5 Department of Periodontology and Implantology, Hitech Dental College and Hospital, Odisha, India; 6 Department of Public Health Dentistry, King George's Medical University, Lucknow, Uttar Pradesh, India; 7 Department of Public Health Dentistry, Teerthanker Mahaveer Dental college & Research Centre, Teerthanker Mahaveer University, Moradabad, Uttar Pradesh, India

**Keywords:** All-on-four, all-on-six, bone preservation, implant stability, stress distribution

## Abstract

The optimal distribution of stress and preservation of bone in implant-supported prostheses remain critical factors affecting the long-term success of full-arch rehabilitations. Therefore, it is of interest to compare the stress distribution and clinical outcomes of the All-on-Four versus All-on-Six prosthetic concepts in full-arch rehabilitation. Hence, a total of 200 participants were divided into two groups, with 100 participants receiving All-on-Four and 100 receiving All-on-Six implants. This study advances knowledge by providing empirical evidence that the All-on-Six prosthetic concept offers better stress distribution, bone preservation and implant stability.

## Background:

The development of dental implants has revolutionized the field of prosthodontics, offering patients a viable solution for edentulism and significantly improving their quality of life [1]. The introduction of fixed prosthetic reconstructions, such as the All-on-Four and All-on-Six concepts, has become increasingly popular due to their ability to provide patients with a stable and durable restoration [2]. These treatment concepts are commonly used in patients with severe tooth loss, where the entire dental arch needs to be rehabilitated with a fixed implant-supported prosthesis [3]. The All-on-Four technique involves the placement of four implants, two of which are placed at the anterior region of the jaw and two in the posterior region at a 45-degree angle. This technique is designed to maximize the use of available bone and provide sufficient support for the prosthesis [4]. The All-on-Six concept, on the other hand, uses six implants placed strategically to distribute the load more evenly across the arch, providing a greater degree of support and potentially offering better long-term stability. While both approaches have demonstrated clinical success, the question remains as to how these different numbers of implants affect the distribution of stress on the surrounding bone and the implants themselves [5]. The primary concern in implant dentistry is the long-term survival of both the implants and the supporting bone structures. Implants, when subjected to excessive stress, can experience biomechanical complications, such as bone resorption, implant failure or prosthetic loosening. Therefore, understanding the distribution of stress around the implants is essential for optimizing treatment outcomes and preventing complications [6]. Stress distribution around dental implants is a complex process, influenced by several factors such as implant design, number of implants, positioning and the prosthetic material used. Previous studies have shown that an uneven distribution of stress around implants can lead to bone resorption, which negatively impacts the longevity of the implant and the prosthesis [7]. The All-on-Four concept, due to its less number of implants, might concentrate stress on the posterior implants, potentially leading to an increased risk of bone loss and implant failure. In contrast, the All-on-Six concept, with its increased number of implants, may distribute the stress more evenly across the arch, potentially reducing the risk of localized bone resorption and implant complications [8]. Recent advances in computer-aided design (CAD) and finite element analysis (FEA) have allowed researchers to simulate and study the stress distribution patterns around implants with greater precision. These techniques offer valuable insights into the biomechanical behavior of implants under various loading conditions [9]. Several studies have employed FEA to evaluate the stress distribution in implant-supported prostheses, yet few have made direct comparisons between the All-on-Four and All-on-Six concepts in clinical settings [10]. Therefore, it is of interest to compare the clinical stress distribution around implants supporting All-on-Four and All-on-Six prosthetic concepts.

## Methodology:

This study was designed as a comparative clinical trial to evaluate the stress distribution around implants supporting All-on-Four versus All-on-Six prosthetic concepts. A total of 200 participants were recruited for the study, with 100 participants assigned to each of the two treatment groups. The study was conducted over a period of 12 months and included both clinical and radiographic assessments. Ethical approval for the study was obtained from the institutional review board and all participants provided written informed consent. Participants were selected based on specific inclusion criteria, including being adults aged 30-70 years, fully edentulous in either the maxillary or mandibular arches or requiring rehabilitation with fixed prostheses. Additional criteria required sufficient bone volume and quality for implant placement, as assessed by cone-beam computed tomography (CBCT) imaging and the absence of systemic conditions that would contraindicate implant surgery. Exclusion criteria included a history of radiation therapy in the head and neck region, severe skeletal deformities, pregnancy or breastfeeding and participation in any other clinical trials within the past 6 months. The study consisted of two treatment groups: Group A (All-on-Four) and Group B (All-on-Six). Participants in Group A received four dental implants placed in the edentulous arch, with two implants placed anteriorly and two posterior implants placed at a 45-degree angle. Participants in Group B received six dental implants placed in a standard arrangement, with two implants placed anteriorly and four implants placed posteriorly, to better distribute the load across the arch. The surgical procedure was performed under local anesthesia by experienced implantologists. Both groups underwent the same surgical protocol, which included preoperative antibiotic prophylaxis. Implants were placed using a flapless or minimally invasive technique, depending on the patient's clinical presentation and bone volume. The implants used were of the same brand and material in both groups, ensuring standardization of implant design and surface characteristics. A healing period of 3-4 months was allowed for osseointegration before the prosthetic phase. The primary objective of the study was to evaluate and compare the stress distribution around the implants in both groups. Stress distribution was measured using finite element analysis (FEA), simulating the mechanical behavior of the implant-supported prosthesis under various loading conditions. The stress distribution was assessed for both static and dynamic loads, including masticatory forces and parafunctional forces such as bruxism. Pre- and post-operative radiographs, including CBCT, were used to assess bone density and volume changes around the implants. Radiographic analysis provided insight into any bone loss or resorption around the implants during the follow-up period. The primary outcome measure was the stress distribution around the implants, quantified through FEA models. Secondary outcome measures included bone level changes, measured using CBCT scans and radiographs at baseline and at 6-month and 12-month follow-ups. Implant stability was measured using resonance frequency analysis (RFA) at baseline, 3-month, 6-month and 12-month intervals. Patient satisfaction was assessed using a visual analog scale (VAS) and a prosthetic evaluation questionnaire. Complications, such as implant failure, soft tissue complications and prosthetic issues, were recorded during the follow-up visits. Data were analyzed using SPSS (Statistical Package for Social Sciences) software. Descriptive statistics (mean, standard deviation and range) were used to summarize demographic and clinical variables. A paired t-test or Mann-Whitney U test was used to compare the stress distribution and bone level changes between the All-on- Four and All-on-Six groups. The significance level was set at p < 0.05. Participants were scheduled for follow-up visits at 3, 6 and 12 months' post-surgery. During these visits, clinical and radiographic assessments were performed to evaluate implant survival, bone level changes and stress distribution. The follow-up period allowed for a comprehensive analysis of long-term outcomes and provided valuable data on the success of the two prosthetic concepts. This study was conducted in accordance with the ethical principles of the Declaration of Helsinki. Participants were informed about the study objectives, procedures, potential risks and benefits before enrolling. Confidentiality was maintained throughout the study and participants had the right to withdraw from the study at any time without penalty.

## Results:

The results of the study were analyzed to compare the stress distribution, bone level changes, implant stability and patient satisfaction between the All-on-Four and All-on-Six prosthetic concepts. Data were collected at three intervals: baseline, 6 months and 12 months post-surgery. The findings showed significant differences between the two groups in terms of stress distribution, bone level changes and implant stability, suggesting that the number of implants plays a crucial role in the performance of implant-supported prostheses. The stress distribution around implants in the All-on-Six group was significantly lower than in the All-on-Four group at both the 6-month and 12-month intervals. This indicates that the additional implants in the All-on-Six group contribute to a more even distribution of stress across the arch, reducing the localized stress on individual implants (Table 1). Bone resorption was observed in both groups, but the All-on-Four group showed a higher rate of bone loss over time compared to the All-on- Six group. The All-on-Six group exhibited minimal bone resorption, which suggests that the distribution of stress in this group may help preserve the surrounding bone structure (Table 2). The All-on-Six group demonstrated a higher RFA score at both 6 and 12 months, indicating better implant stability compared to the All-on-Four group. The improved stability in the All- on-Six group can be attributed to the additional implants, which distribute the load more evenly (Table 3). The patient satisfaction scores for the All-on-Six group were significantly higher than those for the All-on-Four group at both 6 and 12 months (Table 4). This indicates that patients with the All-on-Six prosthetic concept experienced better overall satisfaction, likely due to better implant stability and less stress-related discomfort. The All-on-Four group experienced a higher number of complications, including implant failures and prosthetic issues, compared to the All-on-Six group. The increased number of implants in the All-on-Six group may have contributed to a reduced risk of complications, as the load was more evenly distributed (Table 5). Bone loss percentage was higher in the All-on-Four group, with a greater loss observed at both 6 and 12 months. In contrast, the All-on-Six group had a lower bone loss percentage, which further supports the hypothesis that more implants lead to better stress distribution and less bone resorption (Table 6). The study found that the All-on-Six prosthetic concept demonstrated superior clinical outcomes compared to the All-on-Four concept. Specifically, stress distribution was more evenly distributed in the All-on-Six group, resulting in less bone resorption, better implant stability and higher patient satisfaction. Additionally, the All-on-Six group experienced fewer complications and a lower rate of bone loss. These findings suggest that the All-on-Six concept may offer advantages in terms of long-term implant survival and overall patient outcomes.

## Discussion:

In line with our results, Pandey *et al.* (2023) [10] found through FEA that the All-on-Six configuration showed lower maximum principal stress values on cortical bone and implant components compared with the All- on-Four model under vertical, horizontal and oblique load conditions, suggesting a more favorable biomechanical behavior for the six- implant design. This supports the conclusion that increasing the number of implants helps distribute occlusal forces over a larger bone area, potentially reducing focal stress peaks. Similarly, Jaiswal *et al.* (2024) [11] compared stresses around the All-on-Four and All-on-Six designs with various prosthetic materials in a 3D FEA model. That study reported consistently lower stress magnitudes around implants and cortical bone in the All-on-Six lattice across all tested materials, further indicating that adding implants in the All-on-Six concept improves biomechanical outcomes. Recent work by Kilic and Caglar (2025) [12] also confirmed that the All-on-Six concept showed lower von Mises stress values on implants and principal stress values on bone compared with All-on-Four when evaluating multiple framework materials, highlighting that both implant number and prosthetic material influence stress distribution. While biomechanical modelling often favours the All-on-Six approach for stress distribution, Zhang *et al.* (2023) [13] found no significant difference in long-term implant survival and overall clinical outcomes between All-on-Four and All-on-Six groups in a large clinical cohort, though All-on-Six may offer advantages in specific clinical scenarios (e.g., low bone density or long cantilevers). This suggests that while biomechanical parameters are important, clinical contexts such as prosthetic design, patient anatomy and functional demands also influence outcomes. Gajdhar (2025) [14] highlighted the promising potential of USAG-1 inhibition for regenerating lost dental tissue and stimulating the growth of new teeth. The ability to regenerate dental structures opens up the possibility of enhancing traditional prosthetic approaches, like the All-on-Six concept, with biologically integrated restorations that could further improve long-term outcomes. The combination of regenerative therapies and more stable, well-distributed implant systems may reduce the need for more invasive procedures and help maintain or even improve the bone health of edentulous patients.

## Conclusion:

The All-on-Six prosthetic concept offers superior outcomes in terms of stress distribution, bone preservation and implant stability compared to the All-on-Four concept. Thus, we show the benefits of increased implant number in achieving better long-term clinical results. Future advancements, including regenerative therapies, could further enhance the outcomes of implant-supported restorations.

## Figures and Tables

**Table 1:** Stress distribution comparison (Mean Stress Values in N/mm^2^)

**Group**	**Baseline (N/mm^2^)**	**6 Months (N/mm^2^)**	**12 Months (N/mm^2^)**	**p-value**
All-on-Four	4.5	3.1	3.2	0.002
All-on-Six	4.4	2.5	2.6	0.001

**Table 2:** Bone level changes (in mm)

**Group**	**Baseline (mm)**	**6 Months (mm)**	**12 Months (mm)**	**p-value**
All-on-Four	0.2	0.4	0.6	0.003
All-on-Six	0.2	0.2	0.3	0.004

**Table 3:** mplant Stability (Resonance Frequency Analysis - RFA Scores)

**Group**	**Baseline (RFA)**	**6 Months (RFA)**	**12 Months (RFA)**	**p-value**
All-on-Four	68.5	70.2	72	0.014
All-on-Six	69	74.1	76.3	0.008

**Table 4:** Patient satisfaction (VAS score)

**Group**	**Baseline (VAS)**	**6 Months (VAS)**	**12 Months (VAS)**	**p-value**
All-on-Four	5.1	7.4	7.7	0.005
All-on-Six	5.2	8.3	8.5	0.002

**Table 5:** Complications (number of complications per group)

**Group**	**6 Months**	**12 Months**
All-on-Four	6	8
All-on-Six	3	4

**Table 6:** Bone loss percentage (change from baseline)

**Group**	**6 Months (%)**	**12 Months (%)**	**p-value**
All-on-Four	6.5	9.2	0.012
All-on-Six	4	5.5	0.014

## References

[1] Nayyar A (2026). J. Dent. Health Sci..

[2] Loyola D (2025). Cureus..

[3] Siadat H (2018). J Dent (Tehran)..

[4] Taruna M (2014). J Clin Diagn Res..

[5] Minase DA (2024). Cureus..

[6] Bandela V (2025). Acta Stomatol Croat..

[7] Zanardi PR (2015). J Craniofac Surg..

[8] Soto-Penaloza D (2017). J Clin Exp Dent..

[9] Elsheikh SA (2025). Int Dent J..

[10] Pandey A (2023). J Indian Soc Periodontol..

[11] Jaiswal SB (2024). Cureus..

[12] Kilic S, Caglar I (2025). Int J Prosthodont..

[13] Zhang Y (2023). Clin Implant Dent Relat Res..

[14] Gajdhar SK (2025). J. Dent. Health Sci..

